# Combat Medic-Performed Auscultation Versus Thoracic Ultrasound Image Interpretation for Pneumothorax Detection: Look or Listen?

**DOI:** 10.7759/cureus.68657

**Published:** 2024-09-04

**Authors:** Seth A Brown, Brandon M Carius, Jonathan D Monti, Rachel S Robeck, Darron K Fritz

**Affiliations:** 1 Emergency Medicine, Brooke Army Medical Center, San Antonio, USA; 2 Emergency Medicine, Madigan Army Medical Center, Tacoma, USA

**Keywords:** combat medic, pneumothorax, ultrasound, pocus, military, trauma assessment

## Abstract

Background

Pneumothorax (PTX) is a potentially life-threatening condition encountered by U.S. Army combat medics on the battlefield. PTX, when left untreated, can progress to tension PTX, a leading cause of preventable death on the battlefield which can be difficult to diagnose based on physical exam alone due to variable physical exam findings. Prior literature shows medics can accurately use point-of-care ultrasound (POCUS) to diagnose PTX in cadaver and porcine models; however, no studies have directly compared the diagnostic accuracy of ultrasound (US) and physical exams performed by this population. We sought to compare medic diagnostic accuracy of a simulated PTX using these two diagnostic modalities.

Methodology

We conducted a prospective, observational study in which medics from the Flight Paramedic Program at Joint Base San Antonio-Sam Houston, TX received a standardized 30-minute training on physical exam and US diagnosis of PTX, followed by a 20-minute hands-on US familiarization session. Participants were then randomly selected into cohorts to evaluate 12 high-fidelity manikin lung fields and 12 thoracic US video clips for the presence of PTX in a simulated combat environment. Cohorts evaluated the same manikins and thoracic US video clips but in opposing sequences. Our primary outcome compared the sensitivity and specificity of PTX identification via thoracic US image interpretation and physical exam.

Results

In total, 21 medics evaluated 252 hemithoraces and interpreted 252 thoracic US images. We found a statistically significant difference favoring medics’ sensitivity with US image interpretation over physical exam to detect PTX (85.7% vs. 72.4%, p = 0.004). There was no statistically significant difference in specificity between these modalities (81.9% vs. 69.4%, p = 0.139).

Conclusions

After brief training, medics demonstrated greater sensitivity to detect PTX using thoracic US image interpretation compared to physical exam in a simulated combat environment. Further studies involving medics’ ultrasound image acquisition capability in human patients in austere combat environments are warranted.

## Introduction

Pneumothorax (PTX) is a potentially life-threatening condition involving the collection of air within the pleural space and is a leading cause of preventable battlefield death on the modern battlefield [1]. Left untreated it may progress to tension PTX causing increased pressure on the lungs, mediastinal structures, and the heart which can impair venous return, cause hypoxia, and, ultimately, result in cardiovascular collapse. Early diagnosis, close observation, and treatment are important to prevent this progression.

U.S. Army combat medics historically are taught to clinically diagnosis PTX on physical exam findings without the assistance of adjunct modalities such as ultrasound (US), chest radiographs (CXRs), or computed tomography (CT) [2]. Medics’ acumen in clinical diagnosis without adjunct imaging modalities often misses or inappropriately diagnoses PTX, resulting in both untreated PTX and unnecessary interventions for non-existent PTX [3]. Published studies demonstrate that noise pollution and other common battlefield confounders adversely affect the sensitivity and specificity of clinical diagnosis of PTX by physical exam alone. Hospital-based trauma centers now utilize the thoracic US as the standard of care for screening for life-threatening PTX in the trauma bay [3-6]. There is a small but growing body of literature that suggests that medics and other non-physicians can reliably use point-of-care ultrasound (POCUS) to diagnose multiple conditions, including PTX [7-12]. However, prior literature is largely limited in comparing medic groups between types of didactic instruction, without comparison to traditional physical exams. Therefore, we sought to compare the sensitivity and specificity of physical exams and US imaging interpretation performed by medics to diagnose PTX in a simulated combat environment.

## Materials and methods

The Brooke Army Medical Center Institutional Review Board approved protocol #942357 after expedited review. We conducted a prospective, randomized, crossover study utilizing a convenience sample of active-duty U.S. Army medics stationed at Joint Base San Antonio-Fort Sam Houston, Texas. Volunteers who held a military occupational specialty of 68W (Combat Medic Specialist) were included for participation. Volunteers were excluded if they had any physical limitations that prevented them from participating in the study.

A sample size calculation using online software, JMP version 13.2 (SAS Corp., Cary, NC, USA), determined that 15 subjects would achieve a sensitivity and specificity of greater than 90% with a 10% effect size, an alpha less than 0.05, and a confidence interval (CI) greater than 95%. This analysis was conducted on the premise that each subject would physically examine 12 hemithoraces and interpret 12 US video clips for a minimum of 360 individual evaluations.

Data collection occurred during previously scheduled flight paramedic training. Investigators screened volunteers for exclusion and then provided a 30-minute didactic lecture and 20-minute hands-on US familiarization session. Volunteers received demographic surveys and an answer card to record their responses. Demographic sheets included a participant identifier and were randomized into two cohorts of equal size.

Each subject evaluated 12 hemithoraces on Trauma FX® high-fidelity manikins (TacMed Solutions, Anderson, SC) capable of simulating normal lung field anatomy, physiology, and auscultation. These manikins can also simulate PTX with corresponding absent breath sounds and chest rise and fall. The primary investigator confirmed that all appropriate manikin functions were working properly before starting the testing. The hemithoraces were randomized by an online number generator (www.randomizer.org) for assignment to normal gross chest wall dynamics and auscultation or PTX physiology. Randomization resulted in five lung fields selected for PTX physiology per participant iteration.

Similarly, randomization resulted in five out of 12 US video clips being selected to demonstrate the PTX presentation. All US video clips were obtained using a high-frequency linear probe from a Sonosite® PX US machine (Fujifilm Sonosite, Inc., Bothell, WA) supplied by the Emergency Medicine Ultrasound Fellowship at Brooke Army Medical Center. During testing, investigators simulated a combat environment by playing loud ambient background noise consisting of gunfire, explosions, and aircraft engine/rotor-wash noise. Investigators conducted testing in a non-climate-controlled tent, similar to that of a forward-deployed environment.

The first cohort evaluated the manikins followed by a review of US video clips. The second cohort evaluated the US video clips first and then evaluated the manikins. A study investigator observed each subject throughout their testing and monitored the completion time of each manikin and US clip. The grader observed the participant’s interpretation and prompted the subject for an interpretation at the one-minute mark, if not previously recorded. The subjects recorded each response on their answer sheet; the investigator provided the time-to-completion to the participant to record on the answer sheet as well. After completion of the study, subjects exited the testing facility and were instructed to not interact with other study subjects waiting to participate.

Investigators used the chi-square test to compare the cohorts with statistical significance set at a p-value of 0.05 and a CI of 95%. CIs were calculated using the Wilson approximation. Investigators used online software JMP version 13.2 (SAS Corp., Cary, NC, USA) for data analysis. The t-test was utilized to analyze the time to completion for each answer. A two-way analysis of variance was then conducted to compare the times from each cohort.

## Results

Over three non-consecutive days, 21 medics in the Flight Paramedic program participated in the study. Most were male (n = 17, 81%) in the rank of sergeant (E-5, n = 14, 67%). The participants’ time in service ranged from 3 to 12 years (Table 1). Nine (42.8%) participants reported no previous US experience, six (28.6%) reported fewer than two hours of experience, two (9.5%) reported between two and eight hours of experience, and one (4.7%) reported more than eight hours of experience. No participant reported any formal training on thoracic US. US experience was nearly even between the two cohorts, and each participant completed 12 physical exams and 12 US video interpretations.

**Table 1:** Volunteer demographics. US = ultrasound

Characteristics	Total (%)
Sex	
Male	17 (80.9%)
Female	4 (19.1%)
Age (years)	
18–21	1 (4.8%)
22–25	3 (14.3%)
26–29	8 (38.1%)
30–33	5 (23.8%)
>34	4 (19%)
Rank	
E-3	0 (0 %)
E-4	3 (14.3%)
E-5	14 (66.6%)
E-6	3 (14.3%)
E-7	1 (4.8%)
Prior US experience	
None	12 (57.1%)
<2 hours	6 (28.6%)
2–8 hours	2 (9.5%)
>8 hours	1 (4.8%)

The 12 US video clips were viewed by all 21 study participants resulting in 252 US video interpretations; two US video clips experienced unexpected technical issues that adversely affected the participants’ interpretation. After data collection was completed, the study investigators presented the technical issues to a panel including an external US fellowship-trained reviewer. The panel agreed that the two affected US clips should be excluded, resulting in 210 total thoracic US interpretations, which remained above the sample size calculation threshold of 180 US images.

Across the 210 thoracic US interpretations, medics demonstrated a mean sensitivity of 85.7% (CI = 73-88%) and a mean specificity of 81.9% (CI = 77-91%) for detecting PTX (Table 2). Medic physical exam on manikin simulators yielded a mean sensitivity of 72.4% (CI = 63.2-80%) and a mean specificity of 69.4% (CI = 61.5-76.3). Comparatively, medic-interpreted US yielded a significantly higher mean sensitivity compared to physical exam (p = 0.004) without significantly increased specificity (p = 0.139). Medics responded faster with US than a physical exam, but without significance (12.54 vs. 13.70 seconds, p = 0.235).

**Table 2:** Comparison of sensitivity and specificity for detecting pneumothorax between ultrasound and physical exam. CI = confidence interval

Evaluation modality	Sensitivity (%) (95% CI)	P-value	Specificity (95% CI)	P-value
Ultrasound image interpretation	85.7 (77.8-91.1)	0.004	81.9 (73.5-88.1)	0.139
Physical exam	69.4 (61.5-76.3)		72.4 (63.2-80.0)	

Although there was no significant difference in US sensitivity or specificity with period effect, mean sensitivity was significantly higher with a sequence of US as the first method of evaluation tested compared to the second (90.9% vs. 72.0%, p = 0.01) (Table 3). No significant sequence effect was demonstrated for US specificity. No significantly different time to answer was noted between the cohorts or between different levels of prior US experience.

**Table 3:** Period and sequence effect analysis. PE = physical exam; PE1 = physical exam first; PE2 = physical exam second; US = ultrasound; US1 = ultrasound first; US2 = ultrasound second

Period and sequence effects	US sensitivity	P-value	US specificity	P-value
Period 1 (US1 & PE1)	0.78	0.742	0.77	0.596
Period 2 (US2 & PE2)	0.76		0.76	
Sequence US1 (US1 & PE2)	0.909	0.01	0.80	0.066
Sequence PE1 (PE1 & US2)	0.72		0.64	

## Discussion

After a brief didactic and hands-on training, we found a significant difference favoring medics’ sensitivity with US image interpretation over physical exam to detect findings of PTX. Interestingly, this occurred with an observed sequence effect favoring increased medic US interpretation when performed first compared to those performing physical exams first. This may be due to the stress of continued testing with a less familiar modality being performed second in sequence. We found no significant differences in time to answer, demographics between cohorts, or period effect influences on medic US image interpretation sensitivity or specificity.

Our findings are the first to directly compare US and physical exams for PTX evaluation performed by medics. Prior literature notes that auscultation is traditionally the sole diagnostic means to evaluate for PTX in the pre-hospital environment [5,6]. However, given the noise pollution of combat and patient transport, reliance on auscultation may be inadequate and lead to inaccurate assessment and/or improper management. Conversely, US is not subject to ambient noise interference and therefore can provide improved diagnostic assessment for pre-hospital and en-route care [5-7].

Our findings demonstrate lesser sensitivity (91% vs. 85.7%) but similar specificity (81% vs. 80%) when compared to the only prior research of medic-dedicated US evaluation for PTX by Meadows et al. [7]. While grossly comparable given medic populations and combat-simulated environments, methodology differences in this study compared to ours are notable. Meadows et al. utilized US-naïve medics, defined as those with less than one hour of formal US training. These participants were asked to perform US image acquisition and interpretation on a ventilated cadaver model. Comparatively, our study utilized medics with varied prior US experience to interpret US images acquired by other persons and compared that with physical exams on a simulated model. While this limits direct comparison, it is important to note limitations in the Meadows et al. study when considering our findings.

Meadows et al. used a single cadaver with a posterior thoracotomy incision and a clamped left mainstem bronchus to simulate pneumothorax; the sonographic findings created by this model may not mimic those found in live patients. Conversely, our study design utilized 12 unique US video clips obtained from live human patients with varied pneumothorax severity; this realistically represented varied patient presentations and associated sonographic findings. As such, our participants’ sensitivity may be reasonably decreased given that (1) our participants could not employ image acquisition enhancement techniques, and (2) varied pneumothoraces produce variable sonographic findings. Meadows et al. also did not note the time it took for medics to state their determination of PTX presence, further limiting comparison. Despite these methodological differences, our study’s findings corroborate previous data demonstrating that medics can use US imaging to identify PTX with minimal training.

Prior work by Monti et al, found non-physician medical personnel, including physician assistants, medics, veterinary technicians, and food service inspectors, were able to correctly identify 21 out of 22 pneumothoraces present in a porcine model with 95.4% sensitivity and 100% specificity after brief didactic instruction [8]. However, similar to Meadows et al., this study differed in allowing participants to scan the simulated patient themselves, without specific restrictions or annotation of time to interpretation [7,8]. These differences and the absence of subgroup analysis of medic performance also limit comparisons between the studies. However, similar to studies by Meadows et al. and Monti et al., our findings continue to add to a growing body of literature demonstrating that medics can employ US accurately for diagnosing PTX, as well as other medical conditions. The accuracy of medic-employed US for PTX detection adds to prior literature of medics successfully using US to identify intra-abdominal hemorrhage, fractures, skin abscesses, and foreign bodies [7-12].

This study was primarily limited by the use of manikins to simulate physical exam findings. However, the use of manikins is important to establish a comparison both for future training as well as potential future studies. Manikins are limited by their anatomy, yet the standardization of these training aids is a utility in studies to enable uniformity across testing. However, the accuracy of manikins for PTX simulation is not yet fully established or validated, limiting applicability. As noted, our study participants only performed US image interpretation which does not fully reflect the requisite skills for POCUS to diagnose PTX in an austere environment. POCUS demands clinicians be skilled not only in image interpretation but also in image acquisition and clinical integration of interpreted findings which are skills not assessed in this study. Lastly, the small, select population of medics enrolled in the Flight Paramedic program from a single military installation also may limit this study’s generalizability.

Given these limitations, the positive findings of our study should be added to other literature and used as a catalyst for further research to include a larger sample size and diverse populations. Further, future studies should seek to enable medics to perform US image acquisition themselves and, ideally, should be conducted in a live patient population rather than simulated.

## Conclusions

With minimal training, US Army 68W Combat Medic Specialists demonstrated significantly greater sensitivity to detect the presence of PTX with US image interpretation compared to physical exam, albeit without a significant difference in specificity or response time. This study supports a growing body of literature attesting to medics’ ability to use US for patient evaluation. Further investigation is needed with larger, multi-site populations, and with medics obtaining their own US images for further elucidation of the POCUS concept.
